# The effectiveness of using text and pictograms on oral rehydration, dry-mixture sachet labels

**DOI:** 10.4102/phcfm.v13i1.2646

**Published:** 2021-04-22

**Authors:** Jeanne Heyns, Mea van Huyssteen, Angeni Bheekie

**Affiliations:** 1 School of Pharmacy, Faculty of Natural Science, Western Cape University, Bellville, South Africa

**Keywords:** medication label, oral rehydration dry-mixture sachet, pictogram and text, written medicine information, primary health care

## Abstract

**Background:**

Medication labels are often the only information available to patients after obtaining medication from a healthcare practitioner. Pictograms are graphic symbols that have shown to increase understanding of medicine use instructions.

**Aim:**

To compare the accuracy of the interpretation of medicine use instructions from two different oral rehydration (OR) dry-mixture sachet labels – the control ‘routine textonly’ label and an experimental label with ‘text-and-pictograms’.

**Setting:**

Participants were recruited from waiting rooms in public primary health care (PHC) facilities in Cape Town.

**Method:**

Each participant was required to answer six questions about OR preparation. Response accuracy was determined by comparing the participant’s answer to the actual information written on the relevant label. Afterwards, participants could offer their opinion about the label and ways to improve their understanding.

**Results:**

Of the 132 participants who were recruited, 67 were allocated to the experimental group and 65 to the control group. Only the significant difference between the experimental and control groups for the six questions regarding the label, was recorded for the answer that could be read from a single pictogram (*p* = 0.00) on the experimental group’s label. When asked about this question, more control participants (15/65) found the dosing instruction difficult to understand when compared to the experimental group (1/67). A third of the control participants (22/65) indicated that they could not see or locate instructions on the label.

**Conclusion:**

Text and pictograms on written medicine labels may be an effective tool to aid understanding of medicine use instructions amongst patients attending PHC facilities.

## Introduction

Medication labels are often the only information available to patients after obtaining medication dispensed from the pharmacy or by other healthcare practitioners. Inappropriately designed medicine labels may thus contribute to poor interpretation and improper use of medicines, which could adversely affect patient health outcomes. In particular, people with limited literacy are at risk of not understanding the text-based pharmacotherapy information on medication labels.^1,2^ It is therefore important that the quality and form of the information be at the patient’s level of education.^3^ In addition, the risk for misinterpretation is further exacerbated in patients who cannot read and understand the language of the label.^4^

Pictograms are simple, clear graphic symbols,^5^ representing words or phrases.^6^ Pictograms are sometimes used to represent information about medication, doses, precautions and warnings in picture format.^7^ Its use has shown to support patients’ ability to understand medicine information^8^ and adhere to medication regimens.^9^ South African studies of pictograms on medicine labels and patient information leaflets (PIL) are prevalent^10^ and most evidence (although not exclusively) are available for isiXhosa speaking individuals from the Eastern Cape Province for antiretroviral medicines and human immunodeficiency virus (HIV)-associated opportunistic infections.^11,12,13,14^

However, the fact that South Africa has 11 official languages^15^ of which only two (of which English is compulsory) are legally required to appear on medicine labels,^16^ make pictograms, which is language neutral, an attractive option to attempt to solve this problem. Relatively little information is available for pictogram use in the Western Cape Province with a predominantly Afrikaans speaking (46.6%) population.^17^ This study focussed on a local public health concern, namely diarrhoea in children under 5 years, which spikes during diarrhoea season in Cape Town from November to May.^18^ Preventing dehydration that develops secondary to diarrhoeal disease is essential in the control of mortality from diarrhoeal disease.^19^ As such primary health care (PHC) facilities provide oral rehydration (OR) dry-mixture sachets as first line treatment for diarrhoea to prevent dehydration.^19^ Patients have to reconstitute the dry-mixture with boiled and cooled water at home. As such, the medicine label needs to contain a large amount of information as compared to readymade medicines. Alternatively, patients can make their own dry-mixture from sugar and salt at home.^19^

The purpose of this study was to mimic normal practices of the dispensing of OR dry-mixture sachets at PHC clinics in Cape Town and determine how well participants understood the instructions for use from the medication label only, as this is often the only information available to public sector patients as high workload and high volume of patients at public sector facilities often limit the time for individual patient counselling. The objective of this study was thus to compare the accuracy of the interpretation of medicine use instructions from two different OR medication labels – the ‘routine textonly’ label and a label with ‘text-and-pictograms’.

## Research setting

This study was linked to the service learning programme at the School of Pharmacy at the University of the Western Cape (UWC). The ‘text and pictogram’ OR dry-mixture sachets used in this study was compounded by first year pharmacy students following standard operating procedures for an on-campus practical linked to the first year environmental health service learning programme.^20^ The formulation for the OR dry-mixture was obtained from the Standard Treatment Guidelines and Essential Medicines List for South Africa: PHC level^21^ and the compounding was done as per Good Pharmacy Practice Manual published by the South African Pharmacy Council.^22^ The labeled sachets were subsequently distributed to PHC facilities by second year pharmacy students during their maternal and child health service learning rotation. The PHC facilities for this study were thus selected through purposive sampling, based on their involvement in the second year service learning programme. As such, four public sector PHC clinics located in the Tygerberg sub-district of the Cape Town Metropole in the Western Cape Province of South Africa were selected for the study. These PHC facilities provided services free of charge to local underserved communities with a prevalence of high seasonal incidence of diarrhoeal disease.

### Target population and recruitment

The study population included any patient attending a selected PHC facility on the day of data collection. Participants were eligible for the study if they were 18 years of age or older and spoke English, Afrikaans and/or isiXhosa. Exclusion criteria were: (1) severely impaired vision, (2) hearing problems, (3) too ill to participate in the survey and (4) non-English, non-Afrikaans and non-isiXhosa speaking individuals. Participants were selected by convenience sampling, and as a result of the explorative design of the study, Roscoe’s rules of thumb for determining sample size was used.^23^ These rules suggest that a total sample size of between 30 and 500 are appropriate for most research studies, and in cases where the total sample is divided into subsamples (i.e. in the case of this study, experimental and control), a minimum sample size of 30 for each category is necessary. Subsequently, a convenient sample of 67 and 65 for the control and experimental groups were achieved, which satisfied both rules of thumb.

The study was conducted over a 3-week period in September 2016 at four different PHC facilities. Two trained data collectors recruited potential participants located in the PHC facility waiting areas. The study purpose was explained to the participant, using their preferred language, after which written consent was obtained. At each facility, the study participants were alternatively allocated to either the control or experimental group. Once the participant’s group was assigned, the data collector administered the questionnaire in the participant’s preferred language.

### Data collection

The questionnaire was structured into three parts: demographic information, questions about the preparation and use of the medicine, which required reading from the allocated medication label, and explanatory questions regarding participants’ experience of the allocated label. The first part of the questionnaire collected demographic data such as gender, marital status, age, home language, educational level and the ability to read time from a digital watch.

**FIGURE 1:**
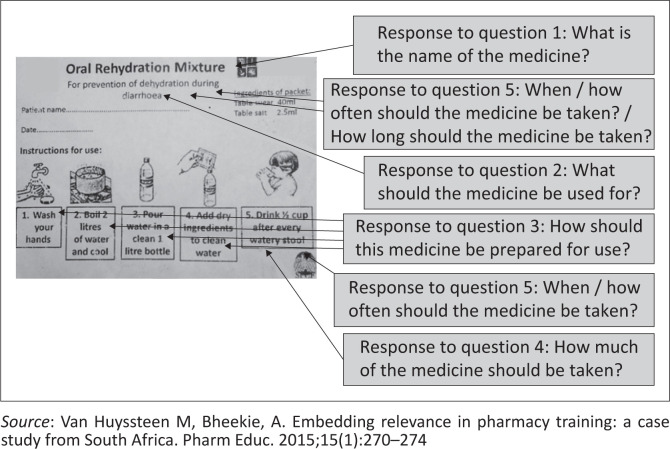
The oral rehydration dry-mixture label mounted on the front of the experimental group (*n* = 67) sachet, packed by University A pharmacy students during on-campus compounding sessions (exact size: length = 5.5 cm, width = 7.5 cm).

**FIGURE 2:**
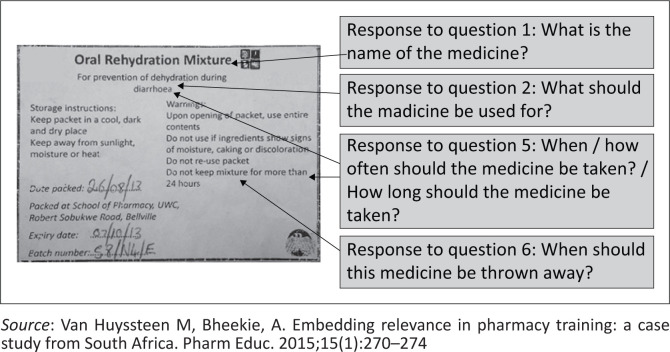
The oral rehydration dry-mixture label mounted on the back of the experimental group (*n* = 67) sachet, packed by University A pharmacy students during on-campus compounding sessions (exact size: length = 5.5 cm, width = 7.5 cm).

**FIGURE 3:**
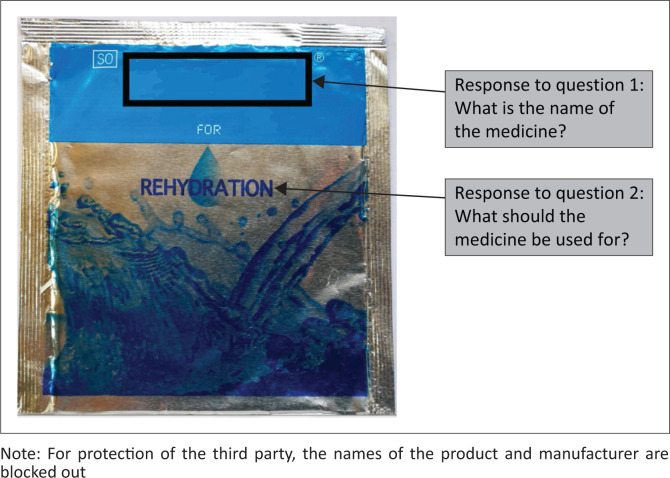
Front of the oral rehydration dry-mixture label of the control group (*n* = 65) sachet that was routinely dispensed at the primary health care facilities (exact size: length = 10.5 cm, width = 10.0 cm).

**FIGURE 4:**
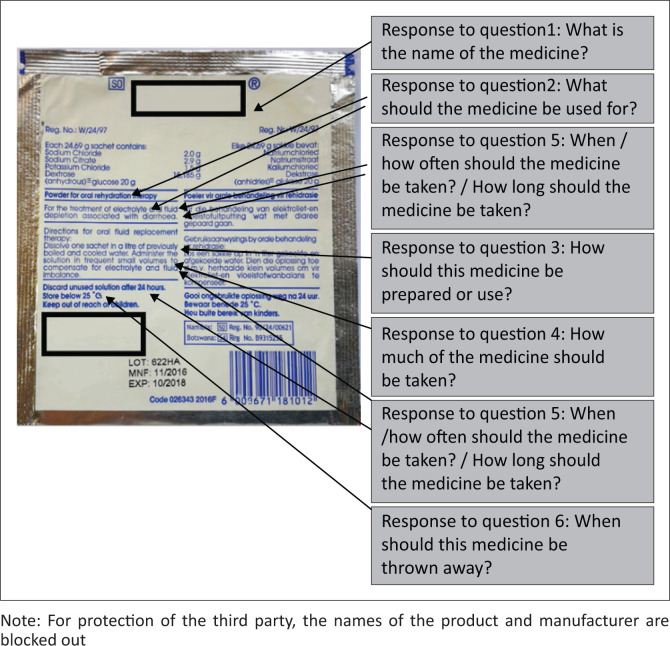
Back of the oral rehydration dry-mixture label of the control group (*n* = 65) sachet that was routinely dispensed at the primary health care facilities (exact size: length = 10.5 cm, width = 10.0 cm).

In the second part of the interview, participants allocated to the experimental group were shown the ‘text-and-pictogram’ medication label (Figures 1 and 2), and the control group was shown the ‘routine text-only’ medicine label that was routinely dispensed at the PHC facility (Figures 3 and 4). The medicine labels were not explained to the participants prior to asking them the following questions about the preparation and use of the medicine:

- What is the name of the medicine?
- What should the medicine be used for?
- How should this medicine be prepared for use?
- How much of the medicine should be taken?
- When/how often and for how long should the medicine be taken?
- When should this medicine be thrown away?

Figures 1 to 4 shows the actual size of the medicine labels and the arrows indicate where the answers were located on the labels.

Part three of the interview involved informing the participants of the intended message of the medication label after which the following questions were asked to probe more about the reasons for the participants’ understanding/not understanding the medicine information and their preferences for the label allocated to them. The questions included:

- Which instructions were easy to understand?
- Which instructions were difficult to understand?
- What do you think could be the reason why you did not understand the instructions?
- Were the pictograms on the label helpful or hindering to your understanding of the medication instructions? (Only for those in the experimental group.)
- What do you think might help you interpret medication labels better? (Only for those in the control group.)
- Finally, participants were asked for any suggestions that might aid their understanding of the medicine label.

### Data analysis

The interviews were audio recorded and transcribed verbatim. Data collectors also made notes on the questionnaires during the interviews. Descriptive statistics were used to describe the demographics of the participants. The demographic profile of the experimental and control groups’ participants in terms of age, education, language and ability to read time will be compared for uniformity of the groups using the Mann–Whitney *U*-test for continuous, and chi-squared test for categorical variables.

The primary outcome of this study was the correct interpretation of the information that appeared on the medicine label. As such, the responses of the six questions relating to the medicine label were allocated to one of three categories, namely: not aligned, partially aligned and fully aligned to the corresponding information that appeared on each label. A score of 3 indicated full alignment with the intended message, and one indicated that there was no alignment with the intended message. Details on the method of scoring for each label are provided in Appendix 1. These categories were compared between the two groups to determine if there were any significant associations between the answer being not aligned, partially aligned or fully aligned and being in the experimental or control group. The chi-squared statistical test was used to determine whether there were significant associations between one of the groups and the alignment of the answer. The statistical post hoc power of association (chi-squared test) was determined retrospectively based the sample size (*n* = 132) on a medium effect size (0.3) and 2 degrees of freedom using G*Power version 3.1.9.2. A power of more than or equal to 80% was considered desirable.^24^

The last part of the questionnaire which recorded participants’ experiences of the allocated label and their explanations of which questions were easy or difficult to answer were coded and thematically analysed. This was done to determine the preferences of participants for the labels.

### Ethical considerations

This research study was explorative and comparative in design. A combination of quantitative and qualitative data was collected from participant interviews using a semi-structured questionnaire. Approval for the study was obtained from the UWC (UA) Biomedical Research Ethics Committee (BM/16/3/01) and the Western Cape Department of Health (WC_2016RP38_657).

## Results

Of the 132 participants who were recruited, 67 were allocated to the experimental group and 65 allocated to the control group. In both groups, two-thirds of the participants (67.0%) attended the paediatric clinic with their children or as caregivers, and the remainder of the participants (33.0%) either sought treatment for themselves or were collecting chronic medicines from the clinic. The time taken for each interview varied between 6 min and 20 min.

### Demographics

The demographic details obtained from the participants are summarised in Table 1. Most (121) participants were women. The median age of the participants was 28 years. Almost two-thirds of the participants (63.63%) reported Afrikaans as their home language. Most (90.15%) of the participants received formal education ranging between grades 7 and 12. Only eight (6.06%) of the participants could not tell the time on a digital watch; of which six were noted to have attained an education level between Grade 7 and 12, and two between Grade 1 and 6. There was no significant difference between the experimental and control groups’ participants in terms of age, education, language and ability to read time.

**TABLE 1:** Demographics and statistical comparability of the two groups of participants.

Variable	Experimental group				Control group				*p*	Overall			
	*n*	%	Median	Range	*n*	%	Median	Range		*n*	%	Median	Range
**Age**, years	-	-	28	18–53	-	-	27	18–56	0.321*	-	-	27	18–56
**Gender**												
Female	59	88.1	-	-	62	95.4	-	-	-	121	92.7	-	-
Male	8	11.9	-	-	3	4.6	-	-	-	11	8.3	-	-
**Education**												
Grade 1–6	3	4.5	-	-	3	4.6	-	-	0.941**	6	4.5	-	-
Grade 7–12	60	89.6	-	-	59	90.8	-	-	0.941**	119	90.1	-	-
Tertiary	4	6.0	-	-	3	4.6	-	-	0.941**	7	5.3	-	-
**Home language**												
Afrikaans	43	64.2	-	-	41	63.1	-	-	0.734**	84	63.6	-	-
English	5	7.5	-	-	3	4.6	-	-	0.734**	8	6.1	-	-
Other	19	28.4	-	-	21	32.3	-	-	0.734**	40	30.3	-	-
**Ability to read time**												
Yes	62	92.5	-	-	62	95.4	-	-	0.718**	124	93.9	-	-
No	5	7.5	-	-	3	4.6	-	-	0.718**	8	6.1	-	-

* , *p*-value calculated from Mann–Whitney *U*-test.

** , *p*-value calculated from chi-squared test.

### Responses to questions regarding the medication label

The results to the six questions are summarised in Table 2. The calculated statistical power was 93%. General trends for the questions show that both groups could identify the name (Question 1) and indication (Question 2) of the medication very well. For the two questions with pictograms on the experimental label, the only significant association between the experimental and control groups were recorded for dosing instructions (Question 4) (*p* < 0.000), which implied that the experimental group was more likely to have a higher number of participants who were fully aligned with the correct answer than the control group. The questions regarding the duration of use (Question 5) and expiry (Question 6) of the medicine was variable for both groups.

**TABLE 2:** Accuracy of responses to the questions related to the medicine label obtained from the experimental group and control group.

Variable	Experimental participants		Control participants		*p*
	*n*	%	*n*	%	
**Question 1: What is the name of the medication?**					
Not aligned	67	11.9	65	7.7	0.407
Partially aligned	67	16.4	65	10.8	
Fully aligned	67	71.7	65	81.5	
**Question 2: What should the medicine be used for?**					
Not aligned	67	9.0	65	12.3	0.820
Partially aligned	67	3.0	65	3.1	
Fully aligned	67	88.0	65	84.6	
**Question 3: How should this medication be prepared for use?**					
Not aligned	67	11.9	65	24.6	0.127
Partially aligned	67	25.4	65	16.9	
Fully aligned	67	62.7	65	58.5	
**Question 4: How much of the medicine should be taken?**					
Not aligned	66	20.9	64	60	0.000
Partially aligned	66	7.5	64	26.2	
Fully aligned	66	70.1	64	12.3	
**Question 5: When/how often and for how long should the medicine be taken?**					
Not aligned	66	23.8	65	40.0	0.154
Partially aligned	66	44.8	65	35.4	
Fully aligned	66	29.9	65	24.6	
**Question 6: When should this medicine be thrown away?**					
Not aligned	66	46.2	65	35.4	0.225
Partially aligned	66	6.0	65	3.1	
Fully aligned	66	46.3	65	61.5	

### Participants’ suggestions to improve understanding

In response to the question, ‘Which instructions were easy to understand?’ almost half (33/67) of the experimental group stated that everything on the label was easy to understand in contrast to only 18.46% (12/65) of the control group. The experimental group also specified instructions depicted by pictograms (38/67) are easy to understand. The rest of the experimental group (14/67) participants mentioned instructions relating to the text to be easy to understand, including small font text surrounded with white space, pictograms and small font text, pictograms and all text, small font text and large font text. The control group mentioned instructions relating to large font text (26/65), small font text (11/65) and bold font text (6/65) to be easy to understand. In addition, almost half of the control group (31/65) found the preparation of medication easy to understand (Question 3).

In response to the question: ‘Which instructions were difficult to understand?’ approximately two-thirds (43/67) of the participants in the experimental group indicated that they did not find anything difficult whilst approximately one-third (22/65) of the control group participants indicated that they did not find anything difficult about the label with routine instructions. More control participants (15/65) reported that the dosing instruction (question 4) was difficult to understand, when compared to the experimental group (1/67). The most difficult instruction for both the experimental (20/67) and control (16/65) participants was reported to be related to the question regarding duration of use (Question 5), which was in text only on both labels.

In response to the question for the reasons why participants thought they might have misunderstood the instructions on the label, a third of the control participants (22/65) indicated that they could not see the information on the medicine label, whereas for the experimental group this was less likely to be of concern (1/67). The second most prevalent reason that both groups (experimental *n* = 10 and control *n* = 9) gave was that they did not understand what was written on the label. Other reasons for not understanding the instructions included not being familiar with the medication, difficulty understanding English, difficulty in reading label information and being illiterate.

The majority of participants (63/67) from the experimental group agreed that the pictograms were helpful for them to understand the label. The control participants provided suggestions on what they thought might have helped them understand the routine label better. A quarter of these participants (16/65) indicated that pictures, larger font size (6/65) and more understandable language (6/65) would have helped them to interpret the medicine label better. Other suggestions included the inclusion of more languages, more information, and verbal explanation from the healthcare provider.

The recurrent suggestion from both groups (experimental 31/67; control 17/65) on how to improve medication labels, was to add pictures. Some participants had no suggestions (experimental 13/67; control 16/65). Participants’ responses could be further subdivided into suggestions to improve the readability of the label and using simpler and more explicit language to improve their understanding of the medicine label. In terms of understanding, experimental participants wanted more clarity on when to take the medicine (7/67), more explicitly specified quantities for preparation and dosage (5/67), simpler language (7/67) and more languages on the label (2/67). In terms of readability, participants suggested bigger font.

In terms of understanding, control participants suggested using different languages on the label (7/65); verbal explanations by healthcare workers (4/65); more explicit instructions on when to take the medication (2/65); what to use the medicine for and to use simple language. Control group participants also had various suggestions of improving the readability of the medicine label such as moving information to the front of the label (3/65), bigger font (3/65), bigger containers, use arrows to show what to do, information should be offered in point form, with step by step instructions, and to use better colour contrasts between the text and its background.

## Discussion

The primary aim of this study was to compare the accuracy of participant’s interpretation of medicine use instructions from two different OR medication labels – the control ‘routine textonly’ label and an experimental label with ‘text-and-pictograms’. This discussion focuses on the participant’s accuracy of interpretation and preference for written instructions on OR dry-mixture sachets regarding the effect that pictograms had on accuracy of interpretation, and in terms of the text on the OR medicine labels.

### Pictograms combined with text on the medication label

Two questions that directly pertained to the interpretation of pictograms and text on the experimental medicine label, included the pictogram pertaining to dosing of OR solution (Question 4) and four sequential pictograms illustrating the preparation of OR solution (Question 3). The question, ‘How much of the medicine should be taken’ was the only question that showed statistical significance in the results with a *p*-value of 0.00. The correct answer appeared as a single pictogram of a little boy drinking from a glass with the text ‘Half a cup after each loose stool’ underneath it. This was the last pictogram in a series of five pictograms on the front of the experimental label. The positive effects of pictograms were consistent with other studies carried out in South Africa where text-only medicine information was compared with text-and-pictogram information.^2,11^

Some participants in this study specifically mentioned the picture of the little boy who was shown drinking the medication on the experimental label. This study did not assess how the boy in the picture was perceived by different cultural and language groups. Pictograms developed for specific cultural groups have been noted to produce higher levels of comprehension and were more likely to be preferred.^3^ When creating pictograms, cultural norms of dress, hair, gestures, facial expressions, objects and buildings should be considered, because some symbols might not be accurately interpreted by other groups.^25,26^

In general, experimental group participants more frequently indicated that everything on their label was ‘easy to understand’ and ‘nothing was difficult’ than the control group participants. In particular, experimental group participants specified that the pictograms on the label were easy to understand and guided their understanding of the label. Indeed, both control and experimental participants suggested pictograms or pictures as suggestions to understand the medicine label better. Similarly, preference for text-and-pictogram presentations to represent medicine information was echoed by participants of a number of other studies.^2,10,11,27,28^

Participants responded more accurately to questions about a single pictogram as compared to a series of pictograms and text. In Question 3 regarding preparation of OR, experimental participants were asked to interpret a sequence of four sequential pictograms and the accompanying text on the label. Experimental participants found it difficult to recall all four steps of the preparation in their answer. This finding supports other studies that found multi-step instructions were confusing to patients.^29^ A sequence of pictograms may be interpreted differently for viewers with low levels of literacy.^25^ Many studies assessing the use of pictograms did not specify the number of pictograms tested.^2,9,11,12,27^

A possible explanation for no difference observed between the groups for the preparation of the medicine (Question 3) might be because of the participant’s prior knowledge. Indeed, almost half of the control group participants (31/65) reported that they found the instructions for the preparation of medication easy to understand even though these instructions were in small and somewhat hidden on the back of the control pack, similar to the dosing instructions. This could be because of a high baseline knowledge of OR solution preparation for both groups as a result of an ongoing OR education programme conducted by the Western Cape Government Department of Health in collaboration with the City of Cape Town, which involved the dispensing of a plastic bottle with instructions for the preparation of OR solution printed in English, Afrikaans and isiXhosa (Personal communication, 16 August 2018). These bottles were dispensed at all the PHC facilities that participated in this study. A person’s existing medicine knowledge, from doctors, pharmacists, packaging design and other public or private sources, affects safe usage of the medicine regimen.^30^

### Text only on medication labels

Medicine labels, or any form of written patient information is commonly evaluated according to two primary criteria, readability and understanding. Readability or legibility relates to the ability of patients to identify/locate/find/acquire specific information on the written document. Readability is a pre-requisite for understanding of the information.

A trend was noted between the good responses for both groups for the name (Question 1) and indication (Question 2) of the medicine as compared to the poor responses for duration of use (Question 5) and expiry instructions (Question 6). On both the experimental and control sachets the name and indication of the medication appeared on the front and back labels, in regular or large font size and surrounded by white space (i.e. no other writing). Similarly, readability has been positively correlated with larger font size,^31^ and using white space and bold font to increase the prominence of the words.^32,33^

However, the relative importance of words on the medicine label has been questioned as research shows that the trade name on medicine labels often receives the most prominence.^34,35,36^ This is problematic as it detracts from the medication’s international non-proprietary name (INN) or generic name.^34^ In South Africa, this is especially problematic, because medicines for public sector are bought by the state on a tender basis, with subsequent changes in the trade name as new tenders are awarded^37^ and in the private sector where medical insurance often only fully covers the generic brand with the lowest cost. More prominence should therefore be given to the generic name of the medication, in a large enough font size for the patient to identify.

In contrast, label (Questions 5 and 6) with instructions appearing on the on the back label, which generally contained a greater number of words and in smaller font, were answered less accurately by both groups. This also aligns with participants’ reasons for not understanding the medicine information, stating that they could not see/locate the information on the label and their subsequent suggestion of using larger font size. Similarly, literature suggests that to prevent patients from missing important information, the readability of text could be improved by surrounding it with white space, using larger font sizes, lists, headers, logical organisation of the information and the use of simple language.^33^ In fact, better readability leads to better understanding of written medicine information.^32,33^

In addition to suggesting strategies for better readability, study participants also suggested the use of simpler and more explicit language on the OR medicine labels. This is in accordance with literature that suggests understanding of health information has been correlated with terminology.^38,39,40^ Even though most of the participants had an education level of grade 7–12 (90.00%), an individuals’ ability to read and understand prescription labels (e.g. ‘administer in frequent small volumes’) may have been significantly worse than their general literacy because of unfamiliar vocabulary (difficult words) and concepts (e.g. ‘administer’ instead of ‘take’, ‘frequent’ instead of ‘often’) on the medication label.^36,40,41^

However readability and subsequently, understandability may be tricky to achieve on OR dry-mixture packs because of the large amount of information required for safe use. Participants also suggested that clinic staff explain the label to patients. Literature agrees that patient counseling is important to provide the intended meaning of text and pictogram instructions on medicines.^2,8,25^ In our study, the OR medicine labels were not explained to participants before asking the participant to interpret the pictograms, because this study aimed to simulate routine practices at PHC facilities where patient counseling is limited.

### Strengths and limitations

This study adds evidence to the effectiveness of pictograms and text on OR dry-mixture sachets in a predominantly Afrikaans speaking patient population. Because of the limited sample size and uniqueness of the study population, the results of the study may not be generalisable to the rest of South Africa. In addition, there was an inherent weakness in the allocation of participants to the two groups, as it was done on a sequential basis rather than a randomised sequence for allocation. Yet, this was mitigated by the well matched groups that were recruited in the end.

The small sample size, also prevented the determination of the influence of language on the interpretation of the medicine label, which is a recognised limitation for the understanding of health information.^38,39,40^ As per regulations, the instructions on the control label were in two of the official languages, Afrikaans and English, and the instructions on the experimental pack were in English only (‘text-and-pictogram’ instructions). Almost all the participants (63.6%) that were recruited for the study were Afrikaans speaking. The Afrikaans speaking participants in the control group might have had an advantage with instructions on the label in their home language, compared to the Afrikaans speaking participants in the experimental group who did not receive instructions in Afrikaans. Alternatively, participants whose primary language was isiXhosa or other African languages might have been at a disadvantage to read the English text on the experimental and Afrikaans and English texts on the control labels. Yet, the effect of these advantages and disadvantages were mitigated by the equal distribution of language between the experimental and control groups.

In terms of the study design, the reader should keep in mind that the comparison was between – (1) an experimental label with pictograms and (English) text, and (2) the text only (in English and Afrikaans) label of the OR sachet that was routinely dispensed to patients at the healthcare facilities. In order to mitigate a possible design bias, the purpose of the research and aim were clearly described and the method was well aligned to try and minimise this. In terms of the method, the two labels were provided and an in depth appendix of how the accuracy of responses was measured was included to introduce transparency in the measurement process. In addition, most literature available on pictograms compares pictogram and text with text only options and these are the results that this study was being compared to. In addition, including text on medicine labels or PILs is essential, and pictograms have been recommended to enhance understanding and not to replace text.

In terms of Question 4, the importance of the effect of the overall differences in the design of the two labels is also important to emphasise. These differences include the pictogram, readability and understandability of the text as well as prominence of the dosing instructions on the two labels. For the experimental label, in addition to the pictogram that participants widely commented on, the text describing the pictogram was in more understandable terms and the overall instruction was in a prominent position on the front label. In comparison the text of the dosing instructions for the control label employed more difficult terminology in particular the terms ‘administer’, ‘frequent’ and ‘volumes’ might have confused participants. In addition, these words had poor readability, because they were in a non-prominent position between other words on the back of the label and in small font, so some participants were unable to locate them on the label. The foregoing explanation illustrates the actual complexity involved in comparing two labels and the problems inherent in focusing on one comparison variable, in this case pictograms and text versus text only. Yet, the purpose of any medicine label should be to effectively confer a minimal amount of information for the patient to use the medicine safely, which justifies an attempt to compare, even though the results might prove to be preliminary at best. Still, these results support best practice label design guidelines and suggest that by combining different aspects of good label design, some labels may become more effective than others.

In addition, because of the nature of the vast information necessary on OR dry-mixture labels, the results of this study should also not be extrapolated to readymade medicines that have less and more simple instructions (i.e. take one tablet 8 hourly). Recommendations of this study should also be limited to OR dry-mixture sachets or other medicines that have to be reconstituted before use.

Another limitation of the study was that, besides asking participants about their highest level of education, no objective health-literacy test was administered to the participants. The most commonly used health-literacy test in a medical setting is the Rapid Estimate of Adult Literacy in Medicine (REALM) tool. However, validation testing of the REALM in developing countries suggests the use of validated test items in local language for reliable results.^42^ In addition, a recent South African study employing the REALM adapted for South Africa showed that although patients did well in the REALM based on word recognition and pronunciation of selected medical terms, it did not translate into how well these terms were actually understood when testing English comprehension.^43^ Similarly, other pictogram studies also tended not to use the REALM^8,9,11,12,28^ or rather used literacy/comprehension tests as reference point for patient literacy.^4,10^

## Implications and recommendations

This study adds evidence to other studies that have confirmed the effectiveness of adding pictograms with text to medicine labels for OR and supports incorporation into practice. However, most medicine labels are small and often do not provide complete information. As such, co-dispensing of a PIL might be an option to improve medicine use. Indeed, the South African Health Products Regulatory Authority (SAHPRA) guidelines for PILs state that ‘pictograms may be used as an additional measure if they make the message clearer to the patient’.^16^ Another important practice implication to keep in mind is that evidence showed that pictograms work best if accompanied by the healthcare practitioner’s verbal explanation. In addition, pictograms that have been developed with feedback from the target audience^25^ and/or pilot-testing of pictograms amongst a small sample of potential users have shown best results.^4^

As pictograms are interpreted differently by different cultural groups,^25^ future studies in the Western Cape could focus on differences in the accuracy of interpretation of pictograms by different ethnic and language groups. Language is an important factor that should be considered in future research when making use of routine medicine labels, especially in populations that are largely Afrikaans speaking. This should be anticipated in sample size calculations in order to draw correlations between participants’ language and understanding of the label.

Future studies could help determine patients’ ability to accurately interpret more than one pictogram when instructions include a sequence of pictograms.

## Conclusion

This study found participants were more likely to accurately interpret instructions for mixing and administering oral rehydration (OR) from medicine labels that included text with pictograms as compared to text only. This study also provides evidence that pictogram sequences may be more problematic for patients to understand than a single pictogram. The use of large font size, bold text and white space positively influenced participant’s ability to interpret text on medicine labels.

## Appendix 1

This appendix provides detail on the process of scoring the accuracy of the six questions to participants relating to oral rehydration (OR) medicine preparation and use. The correct answer of each question was written on either the front or back of the medicine label the participant was given. This appendix should thus be read together with Figures 1 to 4 of the article for a contextual understanding for the intended message on the labels. The accuracy of answers were allocated a score of 3 which is fully aligned with the writing on the label, 2 partially aligned or 1 not aligned with the corresponding writing on the medicine label. Below, the process for each question is discussed.

### Question 1: What is the name of the medication?

For the experimental group, the name of the medicine appeared on the front and back of the medicine pack as ‘Oral Rehydration Mixture’. A score of 3 was allocated for ‘Oral Rehydration Mixture’. A score of 2 was allocated if the participant had an answer that indicated that the name was understood but the exact name as it appears on the packet was not given, for example, ‘Mix for dehydration’. A score of 1 was allocated if the participant did not know the name, or if the answer was not in line with the intended message, for example, ‘I don’t know/know diarrhoea’.

For the control group, the name of the medication appeared on the front and back of the medicine pack with the indication ‘for rehydration’ underneath the medication name, on the front of the packet. The following sentence appeared on the back of the medicine pack amongst other information: ‘Powder for oral rehydration therapy. For the treatment of electrolyte and fluid depletion associated with diarrhoea’. A score of 3 was given when the name of the medication was correctly given. A score of 2 was given if the answer was ‘electrolyte powder’ or if the participant answered that the medication was ‘for rehydration’. A score of 1 was allocated if the participant did not know the name, or if the answer was not in line with the intended message, for example, ‘glucose water’.

### Question 2: What should the medicine be used for?

The indication for use on the experimental medicine pack appeared on the front and back of the medicine pack as ‘For prevention of dehydration during diarrhoea’. A score of 3 was given for ‘For prevention of dehydration during diarrhoea’, ‘for diarrhoea’ or ‘for dehydration’. A score of 2 was allocated if the participant had some aspects of the model answer, for example, ‘for the prevention of diarrhoea’. A score of 1 was allocated if the participant did not know the name or if the answer was not in line with the intended message, for example, ‘to keep the blood and body clean’.

For the control group the indication for use appeared on the front of the medicine pack as, ‘for rehydration’, and on the back of the pack as ‘Powder for oral rehydration therapy’ and ‘For the treatment of electrolyte and fluid depletion associated with diarrhoea’. A score of 3 was given for ‘oral rehydration therapy’, ‘for dehydration’ or ‘diarrhoea’. A score of 2 was allocated if the answer was ‘for the stomach’. A score of 1 was given if the participant did not know or if the answer was not in line with the intended message, for example, ‘If the child cannot take in food’.

### Question 3: How should this medication be prepared for use?

The directions for preparation of the medication on the experimental medicine pack were ‘boil 2 litres of water and cool, pour water in a clean 1-litre bottle, add dry ingredients to clean water’. Pictograms and accompanying text appeared on the front of the medicines pack with the ‘Instructions for use’. A score of 3 was given if the following four steps for preparation were mentioned in the answer by the participant:

- Boil the water
- Cool the water
- Use 1 liter of water
- Add dry ingredients to the water/add content of sachet to water

A score of 2 was allocated if only three of the elements of the model answer were mentioned, for example, ‘boil 2 litres of water and cool, pour water in a clean bottle and add this powder to clean water’. A score of 1 was allocated if the participant had one or two elements of the answer, for example, ‘boil water and add mixture’, if the participant did not know or if the answer was not in line with the intended message, for example, ‘throw in a bottle’.

The directions for preparation of the control medication on the medicine pack were ‘Dissolve one sachet in a litre of previously boiled and cooled water’. A score of 3 was given if all four of the steps for preparation were mentioned in the answer by the participant:

- Boil the water
- Cool the water
- Use 1 litre of water
- Dissolve one sachet in water

A score of 2 was given if only three of these four steps were in the answer given by the participant, for example, ‘use a liter of boiled and cooled water’. A score of 1 was given if only one or two of the four steps were in the answer given by the participant, for example, ‘boil a litre of water’, if the participant did not know or if the answer was not in line with the intended message, for example, ‘use one scoop’.

### Question 4: How much of the medicine should be taken?

The directions for use on the experimental medicine pack for how much of the medicine should be taken were ‘Drink half cup after every watery stool’. A score of 3 was allocated for ‘half a cup after each loose stool’ or ‘half a cup’. If the participant had some part of the model answer, but was not correct in all aspects, for example, ‘half a cup daily’, a score of 2 was allocated. If the participant did not know the answer, or the answer was not in line with the intended message, for example, ‘two cups’, a score of 1 was allocated.

The directions for use on the control medicine pack for how much of the medicine should be taken were ‘Administer the solution in frequent small volumes’. A score of 3 was given for ‘frequent small volumes’ or ‘small volumes’. A score of 2 was given if the participant had some part of the model answer, but was not correct in all aspects, for example, ‘the contents of the sachet’. A score of 1 was given if the participant did not know, or if the answer was not in line with the intended message, for example, ‘a glass two times an hour’.

### Question 5: When/how often and for how long should the medicine be taken?

For the experimental label, there were two possible answers to the first part of this question: ‘When/how often the medicine should be taken’. The first possible answer was, ‘Drink after every watery stool’. The second possible answer ‘Take when you have diarrhoea’ could be derived from the indication for use, ‘For prevention of dehydration during diarrhoea’. There were also two possible answers for the second part of the question, ‘How long should the medicine be taken’. The first possible answer could either have been interpreted as how long to take medicine for the intended use or secondly, how long to take the mixture before it should be discarded. If the question was interpreted as ‘How long to take medicine for the intended use’, the first possible answer would be ‘Take until the diarrhoea has cleared up’ and was derived from the indication for use, ‘For prevention of dehydration during diarrhoea’. If the question was interpreted as, ‘How long to take the mixture before it should be discarded’ the second possible answer would be ‘Take for no longer than 24 h’ and was derived from the instruction ‘Do not keep mixture for more than 24 h’.

A score of 3 was given if at least two of the four possible answers were present in the feedback, for example, ‘Take after every loose stool and discard unused solution after 24 h’ or ‘Take after diarrhoea and stop when the stool is not watery’. A score of 2 was allocated when the participant’s answer contained only one element of the model answer, for example, ‘When the packet is open, you should use it all and not keep it for longer than 24 h’ or ‘Use until the diarrhoea stops’. A score of 1 was given if the participant did not know, or if the answer was not in line with the intended message, for example, ‘Use for 1 week’.

For the control group, there were two possible answers to the first part of this question: ‘When/how often the medicine should be taken’. The first possible answer was, ‘Administer the solution in frequent small volumes’. The second possible answer, ‘Take when you have diarrhoea’, could be derived from the indication for use, ‘For the treatment of electrolyte and fluid depletion associated with diarrhoea’. There were also two possible answers for the second part of the question, ‘How long should the medicine be taken’. If the question was interpreted as ‘How long to take medicine for the intended use’, the first possible answer would be ‘Take until the diarrhoea has cleared up’ and is derived from the indication for use, ‘For the treatment of electrolyte and fluid depletion associated with diarrhoea’. If the question was interpreted as, ‘How long to take the mixture before it should be discarded’ the second possible answer would be ‘Take for no longer than 24 h’ and was derived from the instruction ‘Discard unused solution after 24 h’.

A score of 3 was given if at least two of the four possible answers were present in the feedback, for example, ‘Take frequently and discard unused solution after 24 h’ or ‘Take after diarrhoea until diarrhoea has stopped’. A score of 2 was allocated when the participant’s answer contained only one element of the model answer, for example, ‘Discard unused solution after 24 h’ or ‘Take until the diarrhoea has cleared up’. A score of 1 was given if the participant did not know, or if the answer was not in line with the intended message, for example, ‘Take a spoon three times a day until you finish it’.

### Question 6: When should this medication be thrown away?

The experimental pack indicated ‘Do not keep mixture for more than 24 h’. A score of 3 was given for 24 h, or 1 day. A score of 2 was given if the answer indicated that the participant understood some aspects of the answer, for example, ‘When the diarrhoea has cleared up’. A score of 1 was given if the participant did not know, or if the answer was not in line with the intended message, for example, 2–3 weeks.

The warnings on the control medicine pack indicated to ‘discard unused solution after 24 h’. A score of 3 was given for 24 h, or 1 day. A score of 2 was given if the answer indicated that the participant understood some aspects of the answer, for example, ‘a day or two’. A score of 1 was given if the participant did not know, or if the answer was not in line with the intended message, for example, ‘7 days’.

## References

[1] Barros IM, Alcântara TS, Mesquita AR, Santos AC, Paixão FP, Lyra Jr DP. The use of pictograms in the health care: A literature review. Res Soc Admin Pharm. 2014;10(5):704–719. 10.1016/j.sapharm.2013.11.002 24332470

[2] Dowse R, Ehlers M. Medicine labels incorporating pictograms: Do they influence understanding and adherence? Patient Educ Couns. 2005;58(1):63–70. 10.1016/j.pec.2004.06.012 15950838

[3] Dowse R, Ehlers MS. The evaluation of pharmaceutical pictograms in a low-literate South African population. Patient Educ Couns. 2001;45(2):87–99. 10.1016/S0738-3991(00)00197-X 11687321

[4] Kheir N, Awaisu A, Radoui A, El Badawi A, Jean L, Dowse R. Development and evaluation of pictograms on medication labels for patients with limited literacy skills in a culturally diverse multiethnic population. Res Soc Admin Pharm. 2014;10(5):720–730. 10.1016/j.sapharm.2013.11.003 24355379

[5] Dowse R, Ehlers MS. Pictograms in pharmacy. Int J Pharm Pract. 1998;6(2):109–118. 10.1111/j.2042-7174.1998.tb00924.x

[6] Oxford Dictionaries. Definition of pictogram, 2020 [homepage on the Internet]. 2020 [cited 2020 Feb 27]. Available from: https://www.oxfordlearnersdictionaries.com/definition/english/pictogram

[7] Banstola A. Awareness of pictograms among the undergraduate pharmacy students in a pharmacy college in Karnataka, India: A preliminary study. Int J Pharm Teach Pract. 2013;4(1):1–5.

[8] Joshi Y, Kothiyal P. A pilot study to evaluate pharmaceutical pictograms in a multispecialty hospital at Dehradun. J Young Pharm. 2011;3(2):163–166. 10.4103/0975-1483.80306 21731363 PMC3122047

[9] Braich PS, Almeida DR, Hollands S, Coleman MT. Effects of pictograms in educating 3 distinct low-literacy populations on the use of postoperative cataract medication. Can J Ophthamol. 2011;46(3):276–281. 10.1016/j.jcjo.2011.05.004 21784215

[10] Dowse R, Ehlers M. Pictograms for conveying medicine instructions: Comprehension in various South African language groups. S Afr J Sci. 2004;100(11):687–693.

[11] Mansoor LE, Dowse R. Effect of pictograms on readability of patient information materials. Ann Pharmacother. 2003;37(7–8):1003–1009. 10.1345/aph.1C449 12841808

[12] Mansoor LE, Dowse R. Medicines information and adherence in HIV/AIDS patients. J Clin Pharm Ther. 2006;31(1):7–15. 10.1111/j.1365-2710.2006.00696.x 16476115

[13] Dowse R, Ramela T, Barford KL, Browne S. Developing visual images for communicating information aboutantiretroviral side effects to a low-literate population. Afr J AIDS Res. 2010;9(3):213–224. 10.2989/16085906.2010.530172 25860626

[14] Browne SH, Barford K, Ramela T, Dowse R. The impact of illustrated side effect information on understanding and sustained retention of antiretroviral side effect knowledge. Res Soc Admin Pharm. 2019;15(4):469–473. 10.1016/j.sapharm.2018.05.012 29803539

[15] Pascoe M, Mahura O, Dean J. Health resources for South Africa: A scoping review. Health SA. 2020;25:a1378. 10.4102/hsag.v25i0.1378 PMC7433232 32832107

[16] National Department of Health. Patient information leaflets (PILs) 2014 [homepage on the Internet]. 2014 [cited 2019 Apr 6]. Available from: https://www.sahpra.org.za/Publications

[17] Statistics South Africa. Provincial profile: Western Cape [document on the Internet]. Community survey 2016. Report 03-01-07. 2018 [cited 2020 Jun 15]. Available from: http://cs2016.statssa.gov.za/wp-content/uploads/2018/07/WesternCape.pdf

[18] Groenewald P, Azevedo V, Daniels J, et al. The importance of identified cause-of-death information being available for public health surveillance, actions and research. S Afr Med J. 2015;105(7):528–530. 10.7196/SAMJnew.8019 26428743

[19] National Department of Health. Standard treatment guidelines and essential medicines. List for South Africa: Hospital level paediatrics 2017 edition [homepage on the Internet]. 2017 [cited 2019 Apr 04]. Available from: https://www.knowledgehub.org.za/elibrary/hospital-level-paediatrics-standard-treatment-guidelines-and-essential-medicines-list

[20] Van Huyssteen M, Bheekie, A. Embedding relevance in pharmacy training: a case study from South Africa. Pharm Educ. 2015;15(1):270–274.

[21] Department of Health: South Africa. Standard treatment guidelines and essential medicines list for South Africa: Primary health care level [homepage on the Internet]. 2008 [cited 2015 Feb 6]. Available from: http://www.kznhealth.gov.za/edlphc2008.pdf

[22] South African Pharmacy Council. Good pharmacy practice manual [homepage on the Internet]. 4th ed. 2010 [cited 2015 Feb 6]. Available from: https://www.mm3admin.co.za/documents/docmanager/0C43CA52-121E-4F58-B8F6-81F656F2FD17/00052829.pdf

[23] Roscoe JT. Fundamental research statistics for the behavioral sciences. New York: Holt, Rinehart, and Winston; 1975.

[24] Cohen J. Statistical power analysis for the behavioral sciences. Hillsdale, NJ: Lawrence Erlbaum Associates, 1988; p. 18–74.

[25] Montagne M. Pharmaceutical pictograms: A model for development and testing for comprehension and utility. Res Soc Admin Pharm. 2013;9(5):609–620. 10.1016/j.sapharm.2013.04.003 23680485

[26] Kassam R, Vaillancourt LR, Collins JB. Pictographic instructions for medications: Do different cultures interpret them accurately? Int J Pharm Pract. 2004;12(4):199–209. 10.1211/0022357044698

[27] Thompson AE, Goldszmidt MA, Schwartz AJ, Bashook PG. A randomized trial of pictorial versus prose-based medication information pamphlets. Patient Educ Couns. 2010;78(3):389–393. 10.1016/j.pec.2010.01.010 20153597

[28] Dowse R, Ramela T, Browne SH. An illustrated leaflet containing antiretroviral information targeted for low-literate readers: Development and evaluation. Patient Educ Couns. 2011;85(3):508–515. 10.1016/j.pec.2011.01.013 21306856

[29] Wolf MS, Davis TC, Tilson HH, Bass III PF, Parker RM. Misunderstanding of prescription drug warning labels among patients with low literacy. Am J Health Syst Pharm. 2006;63(11):1048–1055. 10.2146/ajhp050469 16709891

[30] Wilke T, Müller S, Neumann K, Loder T. Does package design matter for patients? Pharm Med. 2011;25(5):307–317. 10.1007/BF03256873

[31] Bernard ML, Chaparro BS, Mills MM, Halcomb CG. Comparing the effects of text size and format on the readibility of computer-displayed Times New Roman and Arial text. Int J Hum Comput Stud. 2003;59(6):823–835. 10.1016/S1071-5819(03)00121-6

[32] Leat SJ, Ahrens K, Krishnamoorthy A, Gold D, Rojas-Fernandez CH. The legibility of prescription medication labelling in Canada: Moving from pharmacy-centred to patient-centred labels. Can Pharm J. 2014;147(3):179–187. 10.1177/1715163514530094 PMC4025884 24847371

[33] Shrank W, Avorn J, Rolon C, Shekelle P. Medication safety: Effect of content and format of prescription drug labels on readability, understanding, and medication use: A systematic review. Ann Pharmacother. 2007;41(5):783–801. 10.1345/aph.1H582 17426075

[34] Prescrire International. Drug packaging in 2016: Marketing takes precedence over public health. Prescrire Int. 2017;26(183):161–165.

[35] Pons ED, Moraes CG, Falavigna M, et al. Users’ preferences and perceptions of the comprehensibility and readability of medication labels. PLoS One. 2019;14(2):e0212173. 10.1371/journal.pone.0212173 30794574 PMC6386266

[36] Lalor D. Medicines labeling. Aust Prescr. 2011;34(5):136–138. 10.18773/austprescr.2011.072

[37] Hoffman JM, Proulx SM. Medication errors caused by confusion of drug names. Drug Saf. 2003;26(7):445–452. 10.2165/00002018-200326070-00001 12735783

[38] Berkman ND, Sheridan SL, Donahue KE, Halpern DJ, Crotty K. Low health literacy and health outcomes: An updated systematic review. Ann Intern Med. 2011;155(2):97–107. 10.7326/0003-4819-155-2-201107190-00005 21768583

[39] Herrera H, Alsaif M, Khan G, Barnes N, Rutter P. Provision of bilingual dispensing labels to non-native English speakers: An exploratory study. Pharmacy. 2019;7(1):32. 10.3390/pharmacy7010032 PMC6473342 30934609

[40] La Caze A. Safer dispensing labels for prescription medicines. Aust Prescr. 2018;41(2):46. 10.18773/austprescr.2018.009 29670311 PMC5895471

[41] Wolf MS, Davis TC, Shrank W, et al. To err is human: Patient misinterpretations of prescription drug label instructions. Patient Educ Couns. 2007;67(3):293–300. 10.1016/j.pec.2007.03.024 17587533

[42] Rathnakar UP, Kamath A, Urval M, Unnikrishnan B, Udupa LA, Shenoy AK. Applicability of the rapid estimate of adult health literacy in medicine–short form among patients attending a university hospital in southern India. Int J Healthc Biomed Res. 2014;3(1):196–205.

[43] Janse van Rensburg Z. Levels of health literacy and English comprehension in patients presenting to South African primary healthcare facilities. Afr J Prim Health Care Fam Med. 2020;12(1):1–6. 10.4102/phcfm.v12i1.2047 PMC7061224 32129648

